# Triple threat: pancreatic cystic lesion presenting with spontaneous hemorrhage is found to harbor three distinct neoplasms

**DOI:** 10.1186/s12957-021-02119-3

**Published:** 2021-01-15

**Authors:** Da Eun Cha, Callie Horn, Michael Passeri

**Affiliations:** 1 Surgery Department, Mount Sinai West and Morningside, 425 West 59th Street, 7th Floor, New York, NY 10019 USA; 2 HPB Surgery and Surgical Oncology, Valley Medical Group, Luckow Plaza, One Valley Health Plaza, Paramus, NJ 07652 USA

**Keywords:** Pancreatic cystic neoplasm, Pancreatic serous cystadenoma, Hemorrhage, Intraductal papillary mucinous neoplasm, Pancreatic neuroendocrine tumor

## Abstract

**Background:**

Pancreatic serous cystadenoma (SCA) is a benign, cystic lesion with an indolent growth pattern. Complications such as spontaneous hemorrhage or malignant transformation from SCA are extremely rare. Our case report describes an unusual presentation of a patient with a previously diagnosed SCA, made unique by the presence of three separate neoplasms in the final specimen.

**Case presentation:**

A 74-year-old male with a previous diagnosis of SCA presented emergently with epigastric pain and non-bilious vomiting. Laboratory results were notable for a hemoglobin of 8.3 g/dl. CT scan of the abdomen demonstrated a complex, solid-cystic mass in the pancreatic head with a large hematoma and questionable focus of active hemorrhage. Surgical resection was recommended due to the risk of malignancy, possibility of re-bleeding, and symptoms of severe duodenal compression. Pancreaticoduodenectomy was performed, and final pathology demonstrated three separate neoplasms: serous cystadenoma, intraductal papillary mucinous neoplasm, and neuroendocrine tumor.

**Conclusion:**

While pancreatic SCA are benign tumors that can be observed safely in the majority of cases, surgical intervention is often indicated in patients with large, symptomatic cysts or when diagnosis is unclear. When undergoing surveillance, it is crucial for both the patient and the care team to be aware of the possibility of rare, but life-threatening complications, such as hemorrhage. Likewise, the possibility of misdiagnosis or concurrent neoplasia should be considered.

## Background

Pancreatic cystic neoplasm (PCN) represents a diverse group of pathologic entities with a wide range of clinical implications—most commonly, serous cystadenoma (SCA), mucinous cystic neoplasm (MCN), intraductal papillary mucinous neoplasm (IPMN), cystic pancreatic neuroendocrine tumor (pNET), and solid pseudopapillary neoplasm (SPEN) [1]. PCN is detected with greater frequency due to the growing ubiquity and high resolution of cross-sectional imaging techniques and endoscopic ultrasound (EUS). Computed tomography (CT) and magnetic resonance imaging (MRI) examinations suggest 2.5–5% prevalence of pancreatic cysts with PCN representing 15–20% of all cysts [1]. Of these, SCA is generally considered benign, only rarely degenerating into serous cystadenocarcinoma (< 1% of cases) [2]. MCN and IPMN, by contrast, have greater malignant potential, with an estimated malignancy risk of 10–50% for MCN and up to 60% for IPMN involving the main pancreatic duct [1]. Differentiating between these PCN subtypes on cross-sectional imaging is desirable, but the differences may not be clearly discernible based on imaging alone. Determining the optimal surveillance interval and the need for invasive testing and operative resection are often complex clinical decisions, especially when the etiology of the PCN is uncertain.

Observation has been favored historically once SCA is diagnosed, as the malignant potential is extremely low. Asymptomatic patients with SCA usually undergo surveillance with serial imaging [3], and resection is reserved for symptomatic patients with SCA or uncertainty of diagnosis [2]. Larger tumors are more likely to cause symptoms like abdominal pain, nausea, and vomiting due to local compression [4]. In rare cases, giant SCA may cause splenic vein compression leading to gastric varices or obstructive jaundice requiring surgical resection [5, 6]. A spontaneous hemorrhage from SCA is extremely rare, with only a few reported cases in literature [7–9], and as such, the threat of bleeding has not significantly influenced treatment or surveillance algorithms.

We present a rare case of a patient who presented with spontaneous hemorrhage from a large cystic neoplasm in the pancreatic head diagnosed as SCA 5 years earlier. The patient underwent pancreaticoduodenectomy, and the final pathology was remarkable for three separate neoplasms: SCA, IPMN, and pNET. We discuss the risks posed by SCA, as well as the importance of structured surveillance once SCA is suspected.

## Case presentation

**Fig. 1:**
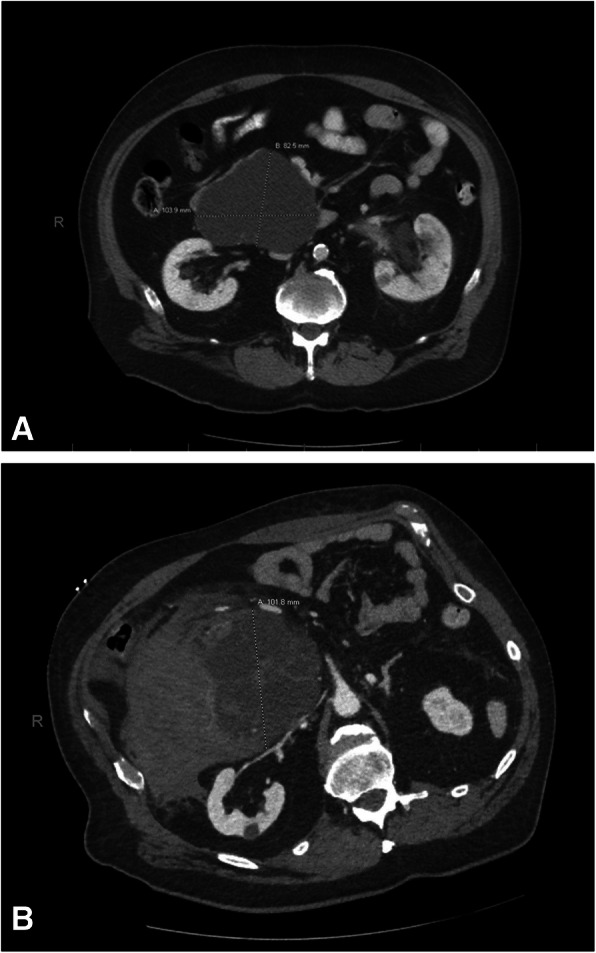
**a** Representative image of CT from 2015 demonstrating 10 × 8 cm septated cystic lesion in the head of pancreas. **b** Image of CT from 2020 demonstrating complex, septated cystic mass now measuring 14 × 6 cm, with focus of active hemorrhage

A 74-year-old male, with a past medical history of SCA diagnosed 5 years prior to presentation, hypertension, nephrolithiasis, and childhood polio with consequent bilateral lower extremity atrophy, presented to the emergency department with epigastric pain associated with persistent, non-bilious vomiting. Patient mentioned a history of a pancreatic cyst, which he had been told was harmless. A CT scan from 5 years prior to his presentation demonstrated a 10 × 8 cm septated cystic lesion in the head of the pancreas (Fig. 1a). In the intervening 5 years, no additional diagnostic work-up or surveillance had been performed.

Upon arrival at the emergency department, the patient was tachypneic, but his blood pressure and heart rate were normal. His abdomen was soft, mildly distended, with generalized abdominal tenderness worse in the upper quadrants bilaterally. Laboratory results were significant for a leukocytosis of 17,000/μL, and a hemoglobin of 8.3 g/dL. A CT scan of the abdomen demonstrated a complex, septated cystic mass in the pancreatic head measuring 10 cm, with a new solid component and an adjacent hematoma measuring 14 × 6 cm, with apparent focus of active hemorrhage centrally. The combined mass-effect of the solid/cystic lesion and the associated hematoma led to a severe compression of the duodenum (Fig. 1b).

Embolization was first considered given the contrast blush seen on CT. However, close interval hemoglobin measurements remained stable and the patient was admitted to the intensive care unit for continued observation.

Several factors and the patient’s clinical status were taken into consideration in determining the next steps in management. The large size of the cystic lesion and the new solid component raised concerns for malignant degeneration [10]. Spontaneous hemorrhage from this lesion and the possibility of a recurrent episode in the near future also imposed a sense of urgency. The presence of persistent symptoms from duodenal compression was also concerning. Thus, the combination of the malignant potential, bleeding risk, and duodenal obstruction led to the recommendation for surgical resection.

The patient was taken to the OR by the hepatopancreatobiliary (HPB) surgery team for a Whipple procedure on hospital day 3. Intraoperatively, a large hematoma was found adjacent to the lesion, extending posterolaterally from the second portion of the duodenum compressing the duodenal lumen. There was no evidence of vascular invasion.

**Fig. 2:**
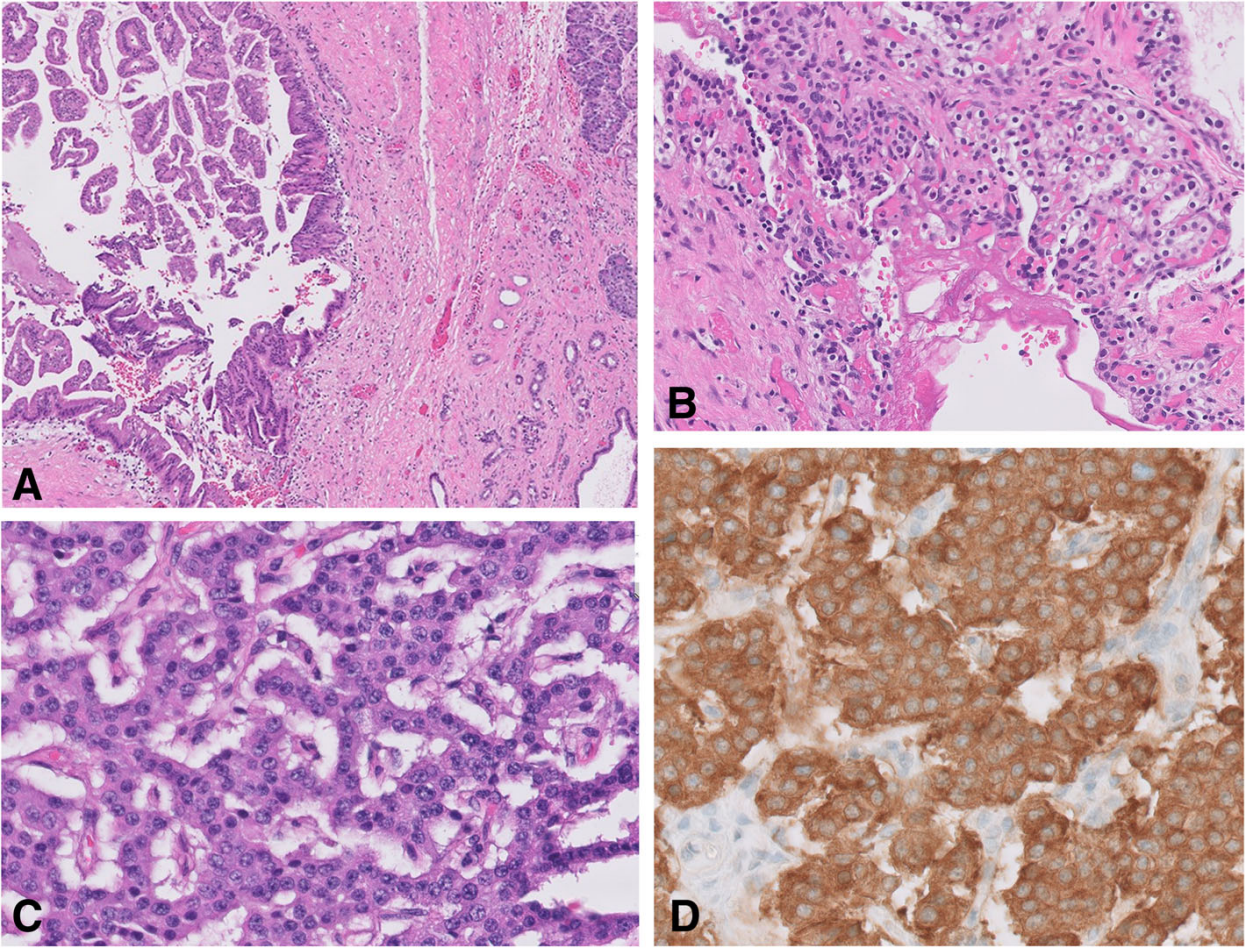
Histology slides of **a** intraductal papillary mucinous neoplasm with adjacent non-neoplastic pancreas tissue (5×), H&E stain, **b** serous cystadenoma (20×), H&E stain, **c** neuroendocrine tumor (40×), H&E stain, **d** neuroendocrine tumor (40×), and synaptophysin immuno-stain

Pathology from the specimen demonstrated chronically inflamed pancreatic tissue and three separate neoplasms. A main duct IMPN (Fig. 2a) was seen arising in the background of macrocystic SCA, which measured 13 × 8 cm (Fig. 2b). The lesion also contained a well-differentiated neuroendocrine tumor (0.4 cm) (Fig. 2c), which was synaptophysin and chromogranin positive and with Ki67 < 3% (Fig. 2d). Pathologists also noted the presence of marked secondary inflammatory and degenerative changes, with prominent necrotizing pancreatitis, fat necrosis, hemorrhage, and fibrosis, accompanying all three lesions. 18 lymph nodes were evaluated, all of which were negative for metastatic disease. The distal pancreatic margin was associated with focally exuberant inflammatory and degenerative changes, including ductal dilation and focal pancreatic intraepithelial neoplasia (PanIN 1).

The postoperative course was uneventful. Patient was discharged on postoperative day 11, delayed due to the patient’s need for extensive physical therapy given pre-existing conditions. The patient has since recovered from the procedure and is doing well. Given the absence of invasive carcinoma, no adjuvant therapy was offered. Surveillance imaging is scheduled 6 months postoperatively.

## Discussion

SCAs are benign tumors that exhibit an indolent growth pattern. They are more commonly found in women, with a median age of 58 years at diagnosis [10]. Many are diagnosed incidentally on imaging studies, as up to 61% of patients are asymptomatic [10]. Risk of malignant transformation to cystadenocarcinoma is reported to be as low as 0.1% and SCA-specific mortality is reported to be nearly zero [10].

Given the benign natural history of SCA, observation of asymptomatic patients is typically recommended. There is currently no consensus on management strategies, with recommendations ranging from surveillance for asymptomatic patients to surgical resection depending on various factors such as SCA size, location, or growth rates [1, 3, 4, 10]. These recommendations hinge on the accuracy of the diagnosis. Distinguishing SCA from other potentially malignant forms of cystic neoplasm with certainty is difficult. One study found that of 2622 cases of resected cystic neoplasm, 60% underwent surgical resection because of uncertainty in diagnosis [10]. Even with improvements in the resolution of cross-sectional imaging modalities, the ability to distinguish between subtypes of PCN has been limited: the accuracy rate for identifying the subtype of PCN is reported to range from 40 to 81% for CT and 40 to 95% for MRI [3]. Certain morphologic patterns of SCA add to the complexity of diagnosis. While 70% of SCA exhibit a microcystic pattern, in which the tumor is composed of numerous, small cysts, some, by contrast, exhibit a macrocystic pattern, which describes fewer large cysts [11]. The macrocystic pattern can resemble MCN or IPMN on imaging [11]. The presence of an intratumoral hemorrhage, as in our patient, also leads to difficulty in differentiation between cystic and solid tumors, since cross-sectional imaging can show heterogeneous enhancement from hemorrhage, necrosis, and cystic degeneration [11].

Endoscopic ultrasound (EUS) can provide supplemental information, especially if fine needle aspiration (FNA) is performed for fluid analysis. Cyst fluid carcinoembryonic antigen (CEA) level has been useful in differentiating between mucinous and non-mucinous pancreatic cysts [12]. Vascular endothelial growth factor (VEGF) has been identified as an accurate SCA biomarker [12]. Carr et al. found that combined CEA and VEGF cyst fluid levels had 95% sensitivity and 100% specificity for the diagnosis of SCA. While these results are encouraging, EUS with FNA may not be technically feasible or may not yield sufficient aspirate for analysis for all patients.

Even when a clear diagnosis of SCA is made based on radiographic and clinical findings, different management strategies have been recommended. The European Study Group on cystic tumors of the pancreas recommends observation for 1 year for asymptomatic patients followed by symptom-based follow-up and surgical resection for patients with symptoms related to compression of adjacent organs [3]. Another single center study on SCA recommends offering surgical resection for symptomatic patients and for patients with SCA measuring ≥ 4 cm, as larger tumors are more likely to become symptomatic and to be associated with faster growth rates [4]. Invasive diagnostic modalities, even resection, are indicated when different types of cystic neoplasm, especially those harboring malignant potential such as MCN or IPMN, cannot be confidently excluded [10].

Our case report describes a patient who developed spontaneous hemorrhage associated with previously diagnosed SCA. Only a few cases of hemorrhage related to SCA have been reported based on our literature search. In two case reports, patients presented with acute abdomen from hemorrhage and underwent distal pancreatectomy of large cysts with a diameter > 10 cm [7, 8]. While rare, hemorrhage associated with SCA may be life-threatening and requires prompt identification and surgical intervention.

Our case report is also unique because the final pathology remarkably identified three different types of neoplasm: macrocytic serous cystadenoma, main duct IPMN, and well-differentiated neuroendocrine tumor. SCAs have been found concurrently with one other type of pancreatic tumors within the same excised specimen, such as IPMN, pancreatic ductal adenocarcinomas, neuroendocrine tumors, and metastatic tumors. In a study of 193 pancreatic serous neoplasms, concurrent neoplasms were found in 13% (27) of cases [13]. Most commonly identified synchronous neoplasms were pancreatic neuroendocrine tumors (6%, 12/193) and IPMN (< 1%, 1/193) [13]. Incidental PanIN was found in 83 cases [13]. Based on our literature search, no prior cases of SCA with two different types of neoplasms have been reported. The possibility of SCA harboring other neoplasms with malignant potential should be considered in outlining a management plan.

## Conclusion

We report the first case of SCA with two synchronous pancreatic neoplasms. While pancreatic SCA are typically benign tumors, the possibility of misdiagnosis or concurrent neoplasia should be considered. When opting for observation, it is crucial for both the patient and the care team to be aware that the tumor may grow, causing local mass effect and eventual symptoms, and that life-threatening complications may arise in rare cases.

## Data Availability

There is no dataset as this is a case report. Data/details of the patient available upon request.

## Abbreviations

SCA: Serous cystadenoma
PCN: Pancreatic cystic neoplasm
CT: Computed tomography
MRI: Magnetic resonance image
MCN: Mucinous cystic neoplasm
SPEN: Solid pseudopapillary neoplasm
HPB: Hepatopancreatobiliary
FNA: Fine needle aspiration
CEA: Carcinoembryonic antigen
VEGF: Vascular endothelial growth factor
PanIN: Pancreatic intraepithelial neoplasia
